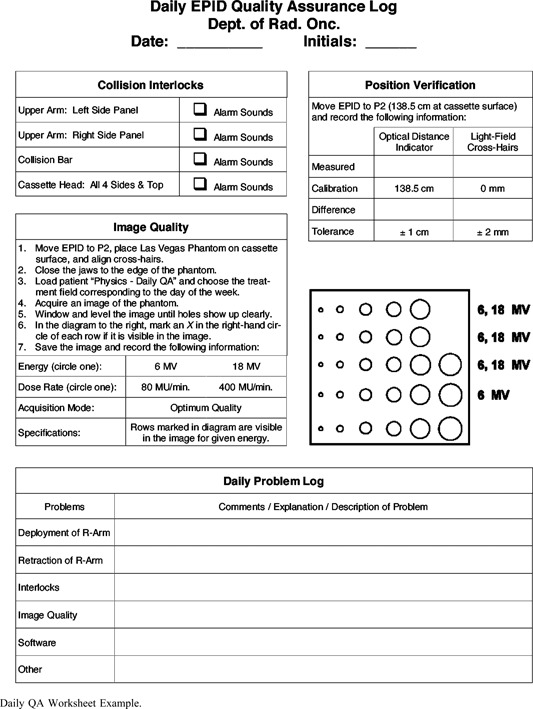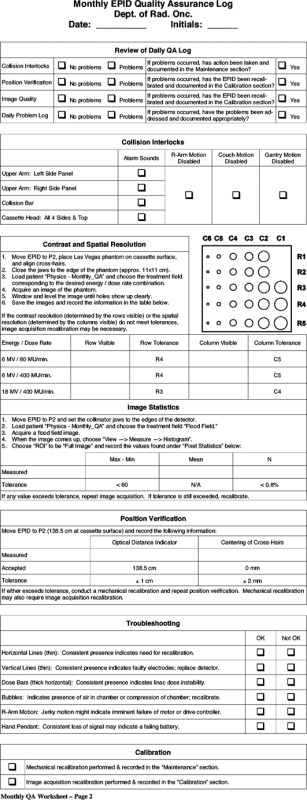# Guide to clinical use of electronic portal imaging

**DOI:** 10.1120/jacmp.v1i2.2645

**Published:** 2000-03-01

**Authors:** Michael G. Herman, Jon J. Kruse, Christopher R. Hagness

**Affiliations:** ^1^ Division of Radiation Oncology Mayo Clinic 200 First Street SW Rochester Minnesota 55905

**Keywords:** portal imaging, electronic portal imaging device, radiotherapy, imaging

## Abstract

The Electronic Portal Imaging Device (EPID) provides localization quality images and computer‐aided analysis, which should in principal, replace portal film imaging. Modern EPIDs deliver superior image quality and an array of analysis tools that improve clinical decision making. It has been demonstrated that the EPID can be a powerful tool in the reduction of treatment setup errors and the quality assurance and verification of complex treatments. However, in many radiation therapy clinics EPID technology is not in routine clinical use. This low utilization suggests that the capability and potential of the technology alone do not guarantee its full adoption. This paper addresses basic considerations required to facilitate clinical implementation of the EPID technology and gives specific examples of successful implementations. © *2000 American College of Medical Physics.*

PACS number(s): 87.53.–j, 87.57.–s

## INTRODUCTION

The benefits of improved treatment technology and three‐dimensional (3D) conformal radiation therapy can only be realized if the target and normal tissues are given the radiation dose prescribed in the treatment plan. Differences between the prescribed treatment plan and the dose distribution actually delivered can compromise or negate the benefits of 3D conformal therapy.

The importance of accurate radiation beam delivery has been discussed theoretically^1^,^2^ and demonstrated clinically.^3^–^6^ The consequences of missing the target, even partially, are a reduction in tumor control probability and an increase in normal tissue complication probability. Significant setup and treatment delivery errors have been reported in film‐based portal imaging studies^7^–^11^ and it has been suggested that an increase in imaging frequency is associated with improved clinical outcome.^12^ Portal imaging using film has become the standard for patient treatment localization.^13^

Film imaging is time consuming, labor intensive and in general, reimbursed for only one port film verification per week. For these reasons, portal film imaging is only practiced once per week per field in most clinics. Therefore, only 20% of the treatments are imaged to verify accurate treatment delivery. The remaining 80% of the treatments rely only on external markers placed on the patient skin and/or an immobilization device. In addition, motion during treatment and day to day variations are not recorded with weekly film imaging. Furthermore, the subjective nature of visual analysis may result in inconsistent conclusions. For these reasons, it has been suggested that an imaging frequency of once per week per field is insufficient to be of value in determining setup accuracy.^14^

The electronic portal imaging device (EPID) provides a more efficient and effective method for determining radiation field placement accuracy. It is capable of capturing images at every treatment and even multiple images during each treatment with little effort. The digital nature of the EPID provides quantitative tools for population‐based or individual patient systematic and random error analysis and replaces the multiple manual steps involved in film imaging (setup, processing, review) with computercontrolled image acquisition, processing, and display.

Over the past 15 years, EPID hardware and software have evolved to the point where computer driven EPIDs can replace film imaging and provide a wealth of information that can be used to reduce errors and improve clinical results. Unfortunately, the power of the EPID and the potential for it to improve the quality of care have not outweighed the obstacles to adopting the new technology.

In this paper, fundamental issues related to the successful implementation of the EPID in the clinic are examined. A custom implementation plan, compatible with each individual clinic and intended use is introduced. Common concerns/obstacles related to routine EPID use are addressed.

Basic qualities of the EPID are discussed, followed by a detailed description of a clinical implementation plan, including quality assurance. This is followed by examples of successful clinical use and a discussion of EPID limitations and costs.

## EPID BACKGROUND

The earliest EPID systems required the presence of a technical expert and were somewhat cumbersome to operate. These devices also lacked the spatial resolution of film, but already demonstrated improved contrast resolution over film.^15^ Conclusions from early work were that the EPID was in general as good as film in delivering localization quality images and better than film imaging with respect to acquisition speed and the potential to use computer aided analysis.^4^,^16^–^20^

### EPID systems

EPID technology will be briefly summarized since it has been detailed in reviews.^21^,^22^ Early array systems used diodes,^23^ scintillators,^24^ or liquid‐based ion chambers.^25^ Early fluoroscopic systems^26^–^28^,^29^ were the precursors of the screen‐mirror systems used today. Present commercial systems will soon be replaced by flat‐panel detector arrays, which offer better resolution and faster response.^30^–^32^

EPID control and analysis software allows efficient and quantitative use of the EPID. The software must integrate hardware manipulation, image acquisition, image processing, image assessment and viewer display. Analysis tools have been developed to automate field edge detection and facilitate manual or computer‐assisted analysis in two or three dimensions, performed either on‐line or off‐line.^33^–^44^ Many of the computer‐assisted tools are aimed at eliminating the inconsistent, qualitative and time‐consuming aspects of visual inspection endemic to film portal imaging, and some of these algorithms are being incorporated into commercial software.

## EPID CLINICAL IMPLEMENTATION

The use of any new technology, even to accomplish a simple goal, cannot be taken lightly. Specific implementation goals, clinical procedures, and protocols for the new technology must be established *before* it can be successfully brought into the clinic. Understanding how the new technology fits into and impacts the clinical process is paramount to successful implementation and long term use. Careful planning ensures that the purchase includes all necessary components and communication peripherals and that personnel commitments can be met.

### Initial preparation for EPID selection

All commercially available EPIDs provide localization quality portal images in less than 3 cGy with the image available for review immediately on a computer workstation. The image detector encompasses up to $30\times 25{\;\text{cm}}^{2}$ field size at isocenter. All the systems are gantry mounted with fixed or variable focus to detector distances, except the latest EPID by Eliav, which is portable and resembles a standard film cassette mount. To minimize interference with patient setup, the gantry mounted systems can be retracted under manual or computer control, or they can be removed altogether. Image acquisition, enhancement, and assessment tools are available from all vendors, with varying degrees of integration. Table I lists examples of questions that should be discussed and answered before EPID implementation. These questions will help a prospective EPID user define his or her goals and needs, and select a system which best meets those demands.

**Table I:** Questions for defining the clinical use of an EPID.

Questions	Options
1. What is he purpose/goal of installing EPIDs in the clinic?	(a) Simple film replacement/routine QA
	(b) Accurate and efficient patient setup and re‐positioning
	(c) Assessment of random and systematic errors in treatment delivery
	(d) Assessment of the efficacy of immobilization techniques
	(e) Inter (between) and intra (within) fraction motion studies
2. For which patients will EPID be used to verify treatment?	(a) All patients?
	(b) Special cases that are difficult to setup?
	(c) Specific disease sites?
3. How will the EPID be used?	(a) Exclusively to eliminate film
	(b) Combined with a predefined port film protocol
4. What will be the frequency of imaging?	(a) Weekly
	(b) Daily
	(c) Dependent on site or patient
	(d) Dependent on the statistics of setup error or decision rules
5. Which image acquisition modes are required?	(a) Single exposure
	(b) Double exposure
	(c) Movie loops
6. What is the choice of reference image?	(a) Digitally Reconstructed Radiograph
	(b) Conventional Simulation film
	(c) First approved EPID image
7. How will image evaluation be accomplished?	(a) Electronically, side by side on computer workstation
	(b) Hard copy on conventional view box
7(a). How many review stations will be needed and at what locations?	(a) At each treatment machine
	(b) Also in viewing rooms
	(c) Also in Physicians offices
8. When will you intervene/adjust setup?	(a) Threshold for corrective action
	(b) On‐line‐intrafraction correction
	(c) Off‐line‐Interfraction correction
9. What image analysis protocol will be used? (This may include image enhancement)	(a) Visual inspection only
	(b) Manual tools
	(c) Semi‐automated
	(d) Automated
9(a). Which analysis tools are available and validated on the system?	(a) Visual inspection only
	(b) Manual tools
	(c) Semi‐automated
	(d) Automated
10. How will physician approval be achieved?	(a) Signed hard copy off‐line
	(b) Electronic signature on‐line
	(c) Electronic signature off‐line
10(a). How will physician comments be communicated to others?	(a) Hard copy
	(b) Electronic annotation within EPID/information system
	(c) Electronic email outside of EPID/information system
11. What are the resources needed for storage, archival and retrieval?	(a) Standalone hard disk
	(b) Distributed database
	(c) PACS
11(a). Is the system DICOM‐RT compliant?	(a) Specific conformance details assessed
11(b). What network and communication infrastructure is required?	(a) No network
	(b) Network with specific bandwidth and security
	(c) Permanent links to Diagnostic Radiology/others required?
12. Implementation of a QA program	(a) Establish baseline mechanical limits and imaging quality
	(b) Establish weekly/monthly protocols
12(a). What are the vendor established QA routines?	(a) How do these compare to our own routines?
13. How will training and education for ALL users be scheduled?	(a) Establish training schedule
	(b) Define personnel responsibilities
	(c) Periodic in‐service to ensure uniformity of clinical practice

**Table II:** Example of personnel requirements for a specific EPID implementation.

Task		Time	per	Personnel	Comment
Acceptance Testing		1–2 days	Installation	Physicist	Additional
Education	Therapist	1 day	Installation	Therapist	per software
	Physician	$\frac{1}{2}\text{day}$	Installation	Physician	revision
Establish QA program		$\frac{1}{2}\text{day}$	Installation	Physicist	
Operation	Imaging	<1−2min.	Tx. Field	Therapist	
	Review	0–5 min.	Tx. Field	Physician/Therapist	Varies between clinics
QA	Weekly	3–5 min.	Week	Therapist	
	Monthly	30 min.	Month	Physicist	
	Quarterly	1–2 hr.	Quarter	Service	

Answering these questions will help prepare the clinic to incorporate the new technology as part of the standard treatment process. These answers should be used to develop EPID system purchase specifications and to understand where additional departmental resources are required.

Table II shows estimates of physician, therapist, and physicist time needed to implement a simple EPID program. It should be noted that practice and responsibilities differ between clinics around the world and these questions and tables should be filled out specific to your needs. Responses to questions 7–10 in Table I can heavily impact personnel requirements.

Table III shows some basic and baseline characteristics of the commercially available EPIDs. In some cases, the manufacturer of the treatment machine may provide an EPID at lower cost and with the most integration to the treatment machine. It is expected that the spatial resolution of the new flat panel detectors will exceed all of the specifications in Table III.

Software tools, information system integration, and ease of data access are changing rapidly and should be addressed in detail. After the questions in Table I are answered, a clear list of specifications should be developed for the vendor. Before any procedures are introduced or modified personnel and resource requirement must be assessed. Only if every member of the team has a good understanding of his/her responsibilities can the new EPID be installed and implemented successfully.

### EPID installation, acceptance, commissioning

Vendor installation of the EPID systems includes mounting the image detector on the accelerator gantry, placing the acquisition and viewing hardware and software at a location indicated by the user and connecting/integrating all the components. This may include interfacing with an information system or PACS, and setting up an image server for the EPID and workstations. Acceptance testing and commissioning allow the user to make certain that the new EPID system meets all performance specifications and is safe to operate in the clinic.

**Table III:** Basic characteristics of commercially available EPIDs. The field size at isocenter is variable for the systems marked by an asterisk. The SDD is source to detector distance. The asterisk indicates SDD is variable. Average resolution calculated from Ref. 45.

Vendor	Elekta	Eliav	Cablon	Siemens	Varian
EPID Type	Video	Video	Video	Video	Ion chamber
Mounting	Rigid	Portable self‐	Retractable	Retractable	Robotic arm
System	removable	contained	adjustable	manually	
Field Size at	25×19	30×25	28×28	30×24 140 cm	25×25
Isocenter (cm)	160 cm SDD	140 cm SDD^*^	140 cm SDD^*^	SDD	140 cm SDD^*^
Software Tools	Available	Available	Available	Available	Available
Average spatial resolution (mm)	2.8	1.6	2.3	2.5	1.9

#### Safety and mechanical

For any EPID system, the integrity of the mounting system and attached hardware should be checked to avoid unexpected dropping of the device. Correct function of the collision detection system should also be checked. In some systems, proper grounding and attachment of high voltage connections should also be verified.

The mechanical integrity and accuracy of the detector mount is important to the quality of EPID data in clinical use. In video‐based systems, the image characteristics may be affected by mechanical imprecision or mechanical defects. In all systems, mounting hardware sag can affect quantitative analysis. Acceptance should include a number of mechanical tests that indicate position reproducibility to within some specified tolerance (usually 2 mm) at a number of gantry angles and detector positions if applicable. Understanding component effects on performance of the EPID system can help the user obtain optimum image quality upon acceptance. Many of these effects have been discussed in the literature and have been addressed by the vendors to improve product quality.^46^–^50^

#### Calibration and dose control

The EPID must be calibrated for various conditions of clinical image acquisition. Depending on the specific EPID, affects of beam energy, dose rate, field size, patient thickness, gantry angle, and detector to patient distance may all require calibration factors for the EPID to operate optimally. In addition, calibration often involves the measurement of a flood or open field image and a background or noise image which are used to remove treatment machine specific influences from clinical images. System specific calibration procedures are available from each vendor, which have been derived from early references.^12^,^29^ Calibration must be performed initially and then checked periodically as part of the ongoing quality assurance program.

Most EPID systems have a dose control mechanism, which allows the amount of dose required to generate an image to be set or adjusted. With some EPIDs, smaller patient doses can be achieved by imaging with fewer monitor units, or decreasing dose rate during imaging. In certain systems, the EPID can turn off the treatment beam after image acquisition is complete. During acceptance, the user should become familiar with and verify the *****system?s dose reduction or beam control features, so they may be facilitated properly in clinical use.

#### Image quality

**Figure 1:**
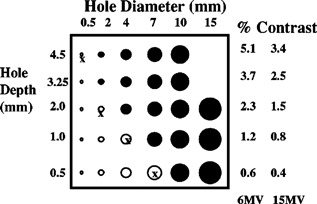
Aluminum Las Vegas phantom for EPID image contrast and spatial resolution. Most EPIDs should be able to resolve all the holes shaded black.

The image quality portion of commissioning examines both spatial resolution and contrast resolution. All present day EPIDs provide 1% or better contrast resolution for larger objects >5 mm. These characteristics are sufficient to perform portal localization on most radiotherapy fields. The Las Vegas phantom (Fig. 1) has been used in acceptance testing and continuing QA.^51^ It is an aluminum block with holes of various depths and diameters. Visualizing a certain hole implies a specific resolution for a given linear accelerator beam. When imaging with a low‐energy photon beam (6 MV) a properly functioning EPID will be able to resolve the 17 holes filled in with black in Fig. 1. Most should be able to resolve another four marked with an X. The newest flat panel detectors are able to resolve all the holes.

Shalev and colleagues have introduced a phantom and software tool that quantifies EPID resolution.^45^ The tool provides quantitative resolution information obtained through a reproducible protocol. The values they report may be used as baseline values for acceptance testing and ongoing QA of any EPID. The user is encouraged to require the vendor to demonstrate that the EPID meets or exceeds spatial and contrast specifications.^45^ Regardless of which phantom is used and whether quantitative software is used, the initial images obtained during acceptance represent base line data for continuing quality assurance of the EPID. They should be stored along with images of anthropomorphic phantoms and other known items (e.g., opaque strips, points) for comparison to later images to check quality. The user is encouraged to include some method of comparing image quality to the base line images in the quality assurance program.

#### Software

Software acceptance and commissioning includes verifying EPID/treatment machine control features, network connections, image maintenance, and image analysis tools. The mode of clinical use and the network environment dictate the necessary procedures. If the system is intended to be used for quantitative patient positioning, then the software must be validated with a series of known transformations. Similarly, image archive and retrieval mechanisms should be checked. Finally, a complete test run with phantom, from simulation through treatment review, can be accomplished to make certain that each step of the process occurs as expected.

### EPID clinical use

A properly installed and maintained EPID provides the treatment team with a tool to perform patient setup verification, organ and target motion studies, compensator design and verification, treatment machine QA and patient dosimetry. It allows more frequent monitoring of patient setup than film imaging and provides computer‐aided assessment of errors. One study suggested that, due to the smaller amount of time needed to image with an EPID, EPID is a more accurate reflection of patient setup error than film. For cases that require rapid setup such as emergent treatment for pain or pediatric patients, the immediate feedback from an EPID is an excellent alternative to film.

### EPID clinical protocol (step by step)

**Figure 2:**
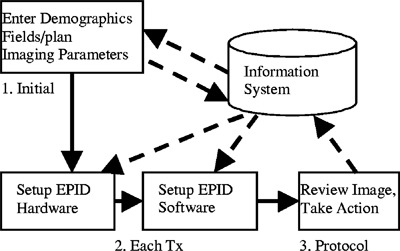
EPID imaging steps 1,2,3.

Figure 2 is a schematic of a simple process for implementing EPID in the clinic. The solid arrows show the steps involved in the process, while the dotted arrows demonstrate the flow of data to and from a fully integrated information system.

(1) At the beginning of a patient's course of treatment, demographic and field data are entered. Image acquisition data is also entered, e.g., single or double exposure, movie loop, etc. The type and amount of data necessary varies depending on the EPID manufacturer. (2) At treatment time, the EPID is put into imaging position, the patient is selected, the field is selected and acquisition parameters loaded. (3) The patient is imaged, and the therapy team responds to the image relative to a predefined protocol. The action may be on‐line or off‐line setup correction or to do nothing. If the EPID is part of an integrated information system, steps 1, 2, and 3 may be simplified or automated.

Image acquisition modes common to all EPIDs include single image, double exposure, and multi‐image movie loop. Image enhancement filters are also standard on all systems, some of which can be activated automatically.

### Error detection and correction strategies

Treatment setup verification can be divided into verification of the geometric configuration of the treatment unit and verification of the patient and target position with respect to the treatment geometry. Both of these become more important and complicated when treating with high doses and 3D intensity modulated radiation therapy (3D IMRT). Correct evaluation of treatment setup involves relating the field aperture and anatomy in a portal image to that in a reference image, and choosing a course of action to reduce any errors present. Understanding what type of error is being analyzed is important for making the proper decision. The basic error types have been summarized:^52^ (1) group deviations, which represent a systematic error that is identical for a group of patients (e.g., a mechanical error in the treatment machine or simulator); (2) systematic error, which is identical for all treatment fractions of a single patient, but not correlated with other patient's errors; (3) random errors or daily variations which are different for each RT fraction of a single patient; and (4) intra‐fractional deviations which are errors caused by movement of the patient during a single fraction. Patient setup errors may have a systematic component due to either equipment or protocol and a random component due to organ motion or daily positioning. With an EPID, the user can obtain enough information to confidently assess the character of errors for simple or complex 3D variations. Only with this data can a proper correction be made, leading to eventual therapy success.

**Figure 3:**
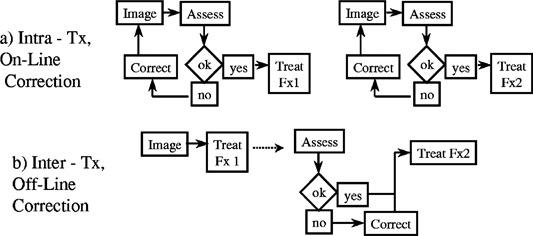
Schematic flow of on‐line vs off‐line EPID correction strategies.

EPID use for patient setup verification and correction can be separated into two general categories, on‐line or intrafractional and off‐line or interfractional. On‐line correction means that a pretreatment port is captured and reviewed. Any corrections are applied before treatment continues [(Fig. 3a)]. Localization portal films are an example of an on‐line correction. The most basic manifestation of off‐line correction is the weekly port film, when the image is examined after treatment, and if necessary, a correction is made at the following treatment session [(Fig. 3b)].

Off‐line correction has also evolved into strategies whereby multiple periodic images are evaluated to improve statistical certainty for one or more corrections over an entire treatment session. Available EPID software provides automatic field edge detection, digital rulers and in many cases manual or automated registration between a portal and reference image.

#### On‐line EPID protocols

**Figure 4:**
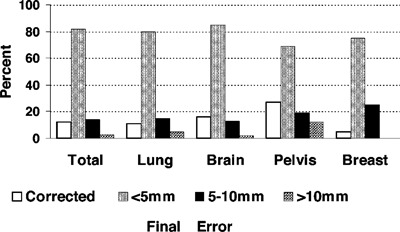
On‐line correction error through visual analysis and final error. Modified from Ref. 18 with permission from Elsevier Science.

An early group of on‐line EPID studies involved taking prospective on‐line action based on visual analysis of a pre‐treatment port. This type of protocol allows the reduction of total setup errors for each individual patient, but can not differentiate between systematic and random components. This procedure has been implemented in a number of centers as routine protocol.^18^,^53^–^55^ Results of these studies indicate that up to 50% of initial fields are judged in error and corrected. The error correction rate is anatomical site dependent and due to visual analysis, observer dependent. While these studies demonstrated improvement in setup accuracy, final off‐line analysis shows that some residual setup error remained. An example of on‐line setup correction and final error is shown in Fig. 4.^18^ The weakness of these protocols is that they depend primarily on two‐dimensional qualitative analysis. The problem with subjective analysis is also presented in Fig. 4, where even after correction based on visual analysis, subsequent off‐line analysis found that a significant number of setups were still in error by more than 5 mm. In addition, manual patient setup correction can increase treatment time. For these reasons, daily on‐line EPID imaging is not practiced in many centers. There are still examples of on‐line correction strategies in use today, where the clinicians feel that the additional time to make a correction is warranted in certain protocols.^56^ This could certainly be the case for 3D IMRT, where high doses and complex setups are common or with patients that are difficult to position.

More quantitative on‐line daily approaches have been developed, which utilize automated EPID analysis tools to substantially increase accuracy, requiring minimal analysis from the therapists. On‐line error analysis has been demonstrated for pelvic and thoracic treatment setup.^57^,^58^ While these studies showed a significant improvement in setup accuracy, additional treatment time was required for patient adjustment as all detectable errors were corrected. An important lesson from these studies is that using the EPID to image more frequently often leads to the frequent detection of errors, or, the closer we look, the more we see!

The computerized nature of the EPID allows it to be integrated into a larger scale decision‐making system. Such an integrated system can help the users decide when it is appropriate to make a correction and when not to, based on physician and treatment planning guidelines established.^59^ The quest for optimization and automation of EPID image analysis is ongoing both by research groups and vendors.

#### Off‐line EPID protocols

Off‐line EPID protocols can be separated into three groups, simple off‐line correction (film model), monitoring and statistical decision models.

*Simple Off‐line*: The EPID can be used exactly as film and a full resolution hard copy can be generated identical to routine practice with portal films. Even in this simple mode, the EPID provides additional benefits compared to film; imaging time is faster and image enhancement (e.g., contrast enhancement, edge enhancement) algorithms can be immediately applied to acquired images. With software analysis rather than hard copy review, error detection can be accomplished manually, with computer assistance in an interactive mode, or in certain cases via fully automated means.

*Monitoring*: The earliest clinical EPID studies were of the monitoring type, where images are acquired, but no action is taken. These efforts demonstrated the ability of these devices to acquire a large amount of information on the clinical practice of radiotherapy. Lam described the frequency and magnitude of field placement errors (FPE) in thoracic and abdominal radiotherapy, suggesting that errors exceeding 1 cm were not uncommon and that conventional planning margins may not be sufficient.^60^ Others have created summary data showing the cumulative effect of daily FPE on the course of radiotherapy for individual patients^61^,^62^ and then extended the methodology to indicate the implications of FPE on treated doses showing increased penumbra at the field edges due to the FPE.^63^ Monitoring has shown that patient setup error can increase or decrease during the course of therapy and that routine imaging is essential to maintain accurate treatment.^64^

**Figure 5:**
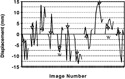
Movie loop data showing displacement of chest‐wall lung interface imaged 6 times per fraction for 11 fractions. Arrows represent daily imaging and W represents weekly imaging.^71^

The EPID facilitates monitoring target and normal tissue motion between and during treatment fractions through multiple image movie loops. These investigations show interfractional (between) and intrafractional (within) motion of critical organs such as the lung and heart in tangential breast treatments or the prostate gland and pelvic anatomy during pelvic treatments. This method has been used to investigate the reproducibility and accuracy of tangential breast field placement.^65^–^67^ The comprehensive analysis enabled by EPID use shows the magnitude and frequency of setup and motion errors for a group of patients, and more importantly for individual patients. This is done with great statistical certainty, recording in excess of 150 images per field throughout treatment of a single patient. An example of motion of the lung‐chest wall interface during tangential breast treatment is shown in Fig. 5, indicating the wide range of motion that occurs due to respiration during treatment, as measured with an EPID. Daily and weekly imaging samples are also indicated in the figure, showing the improvement in statistics when using multi‐image EPID acquisition. This data shows clearly that the statistical sample of motion obtained by weekly portal imaging is almost useless. Tissue motion due to respiration can exceed 2 cm during tangential breast treatments.^68^ This can adversely effect normal lung volume treated and lead to a possible increase in complications.

Another focus of movie loop and motion monitoring studies has been prostate motion. EPID imaging allows multiple images on every fraction, with suitable resolution to visualize radio‐opaque markers in prostate tissue. These data show that while the prostatic tissue relative to bony pelvis does not move appreciably during treatment, it can move over 1.5 cm relative to the bones between fractions.^69^ Other pelvic setup studies show that setup errors exceeding 1 cm were not uncommon, and that these intertreatment values exceed any intrafractional motion errors for the pelvis.^70^

Monitoring studies demonstrate the power of EPID technology to acquire sufficient image data during treatment to *benefit the individual patient.* Analysis of these data allows assessment of institutional technique and patient specific errors that can not be obtained with film. Movie loop techniques may be very useful for monitoring IMRT devices/treatments.

#### Statistical models/decision rules

Statistical methods provide the benefits of basic on‐line correction protocols, without a large increase in time or cost for the information. Maintaining tight planning margins in 3D and IMRT treatment will require intelligent use of an EPID.

*Decision Rule example 1* (analysis based on a global standard): A systematic error correction protocol has been reported that performs imaging and correction based on population error statistics and computer simulation.^72^–^74^ Systematic error of the patient setup is evaluated with respect to a site‐specific population or global standard and if necessary corrected. These studies have demonstrated that reduction of systematic error of roughly a factor of 2 (compared to uncorrected) is achievable, with an average of less than 10 EPID measurements and approximately 0.5 corrections per patient treatment course. In other words, with about the same imaging effort as film, and the tools of the EPID, significant error reduction can be achieved.

*Decision Rule example 2* (analysis based on an individual standard): The ability to gather enough data to make systematic and random error assessment for individual patients with EPID has also been introduced. This allows the field margins and any corrections or modifications to treatment to be patient specific.^75^,^76^ Similar work in the use of EPID for early error detection and correction for dose escalation protocols is also underway.^77^

### Special EPID applications

The utility and efficiency of EPID imaging has been documented in a number of special cases. These include megavoltage simulation and treatment of the obese patient^78^ and efficient placement of lung blocks for total body irradiation.^79^,^80^ Investigators have used the EPID for the design^81^–^83^ and verification^84^,^85^ of compensating filters. Most of these procedures would either be much more time consuming or impossible to perform by conventional means.

The EPID has also been put to use for quality assurance of treatment machines^86^ and of treatment techniques, such as radiosurgery^87^ and dynamic wedge and MLC therapy.^88^,^89^ In each case, the EPID has allowed more precise, quantitative results to be obtained with much less effort than would have been achievable using conventional QA tools. In 3D and IMRT treatment, the EPID could be a critical component of a validation and verification process.

### Patient dosimetry

While setup error and patient motion are quantified with EPID imaging, the ultimate value of concern is dose actually delivered to target and normal tissue. The computer‐generated treatment plan is only an estimate of the dose distribution. Efforts to determine and quantify (*in vivo*) dose in two and three dimensions are underway. The earliest works investigated the characteristics of the various EPIDs for transmission dose measurement.^90^–^94^ These studies indicate that with the proper calibration and care, the EPID can be used to generate an exit dose image within 2–5 % of expected values. Additional work has gone into the interpretation of the EPID image in terms of a quantitative exit dose and implications for dose at the target.^95^–^98^ This type of EPID application can be an important verification instrument in the analysis of 3D and IMRT protocols.

## EPID QUALITY ASSURANCE

The need for quality assurance is well established for any procedure or device used in clinical practice. Various parameters can affect image quality and functionality of EPIDs depending on the type of system. It is important to establish a specific QA protocol to monitor EPID performance at a regular, defined frequency. Mechanical instability can reduce image quality and perhaps render the EPID useless under certain circumstances. For video‐based systems, optical component alignment and proper adjustment is critical. The consequences of a poorly maintained or poorly setup EPID are wasted time, unacceptable image quality and ultimately rejection of the system and EPID technology. Hardware and software parameters and settings must be monitored for proper setting/function. Most QA for the EPID involves image contrast and spatial resolution. Methodology and phantoms for these procedures have been discussed and include developing baseline images and performance data for routine comparison.^45^,^99^ The image and performance data recorded during acceptance and commissioning are the reference standards for continuing QA tests. The QA program must also consider mechanical and safety aspects of the EPID, especially for those using computer controlled and detachable mechanical components.^100^ The vendors are expected to provide guidance for users regarding the QA of the EPID to maintain optimal performance and good clinical utilization.

Weekly or daily tests are concerned with safety interlocks, mechanical stability and basic image quality checks which are usually carried out by the therapists. The QA process also includes reporting any difficulties with operation and quality of the EPID to physics or engineering staff. The monthly EPID QA procedure involves more quantitative performance assessments and checks. In additional to mechanical and safety checks, contrast, noise and spatial resolution have action limits defined. Many simple image quality tests can be done with custom software such as PIPS (Ref. 45) or by using standard phantoms and EPID software. The user is responsible for defining the protocols and action limits used. The weekly and monthly QA tests can be completed in minutes and are an important part of maintaining a clinical EPID. Specific examples of QA worksheets are shown in the Appendix.

Regarding software QA, the accuracy and validity of any clinical algorithm must be tested and documented. This may include testing the software tools with known error and resolution conditions similar to acceptance and commissioning on an annual basis, or when software upgrades are performed.

## EPID LIMITATIONS

Even with the promise and the power of the EPID, some limitations related to hardware, software and integration remain. EPID image detector size spans no more than a $30\times 25{\;\text{cm}}^{2}$ field at isocenter for most commercial systems. Imaging a larger field would require the ability to acquire multiple images with the detector in different locations and digitally add them together. This function is not yet commercially available. The deployment mechanism can be inconvenient and may interfere with patient setup. The worst must be removed completely from the gantry mount for patient access and the best retract completely under computer control.

A second and very important limitation of current EPIDs is the slow development and introduction of standard analysis tools for clinical use in commercial systems. This forces the user to accomplish setup analysis primarily visually or using very basic tools, as has been done with film. Without quantitative analysis, EPID imaging suffers from many of the same drawbacks as film imaging. Developing standards is necessary for the broad and effective use of analysis tools. Most software available commercially also only allows the user to perform two‐dimensional analysis of three‐dimensional setup errors, which can be misleading if out‐of‐plane rotations are large. Reliable automated 3D analysis tools are being developed.^40^,^41^,^44^ The availability of EPID tools for rapid and accurate assessment of patient setup and field placement errors represents an important improvement over film imaging.

The integration of the EPID into a comprehensive information system communicating with the treatment machine, the treatment planning system and image analysis workstations is not yet complete. Most systems require technical experts in the clinical setting to oversee these operations. The EPID system must become an integral part of the treatment system, just as the multileaf collimator has become. Such a system has been described for computer controlled radiotherapy.^101^ The DICOM RT image object has been designed to facilitate storage, communication and retrieval of EPID data as well as other RT imaging data.^102^ This standard should be adopted and supported so that integration remains a possibility with the overall information system.

Finally, hardware integrity and service is still sub‐optimal. Retrofit EPID systems are more likely to suffer from small mechanical and electronic problems. The user and in‐house staff can become frustrated with the apparent high degree of complexity to operate and service the EPID. In many instances, the commercial service personnel are not well versed in the mechanism and repair of the EPID. Better integration and more complete training and education are required to help remove the frustration.

## EPID COST

The major expense for an EPID is the initial cost. Commercially available systems cost between $70,000 and $400,000. The initial expense of a system to accomplish film portal imaging is only about $20,000. However, the ongoing costs for film portal imaging are substantial, while with the EPID, ongoing equipment and per image costs are almost negligible. The EPID also demonstrates savings over film imaging in personnel utilization. The extra amount of time needed to process film and display it for review is expensive, but varies depending on location and who performs the work. It has been shown that for large centers, or smaller centers that image frequently, EPIDs can be more cost effective than film.^103^ If an EPID is expected to last 5–7 years, then film and EPID become approximately equal in overall and per image cost at 4000–5000 images per year. This estimate considers capital costs, annual maintenance, and personnel time for a typical treatment machine treating 250–300 patients per year and imaging 15–20 ports per treatment course. If a center chooses to image more frequently using the EPID, the cost per image can be less expensive than film imaging. In addition, computer‐controlled EPID systems should be more efficient and better integrated, providing a more competitive alternative to film. It is important to note that this cost analysis treats EPID and film as identical in clinical value, ignoring the fact that the EPID provides far more capability than film for imaging and error analysis and in some cases can do things that can not be done with film.

The costs associated with personnel commitment to the EPID process should also be considered. Quantifying potential personnel costs may help prepare the clinic for any changes required in implementing EPID. This could be especially true for 3D IMRT, where implementation resource costs are not small. Depending on the mode of EPID use, resource costs for acquisition, review and maintenance (QA) can be assigned to the physician, therapist or physicist relative to that procedure. An example of initial setup and operation was indicated in Table II. Additional clinic and protocol specific resource assessments must be made and appropriate expectations developed for ongoing use of an EPID. This could include a review of the questions asked in Table I, with appropriate resource allocations assigned.

## CONCLUSIONS

The EPID has come a long way in the last decade, and there is still work to be done. EPID technology is available for clinical use not only to replace film, but it has been shown to be an important tool for setup error assessment and quantification of motion during treatment, determining delivered treatment doses, treatment technique QA and treatment machine QA. Even when EPID is used like film, it has the advantage of faster and more quantitative results. In the case of multi‐image, intrafractional assessment, or quantitative analysis of 3D treatment setup parameters, EPID is the only method to perform the task. The improvement in treatment outcome anticipated with 3D IMRT can only occur if the dose is delivered as planned. The EPID can play an important role in verification and assessment of these complex radiotherapy treatments.

In spite of the many documented successes, EPIDs have not yet received full acceptance in the clinic. This is due to lack of experience and education, hardware integrity, the commercial availability of software tools and full integration into the treatment information system. The capability and utility of EPID systems will continue to improve as better detectors and new software are developed. Present day EPIDs can be successfully implemented in the clinic. The treatment team must establish specific goals for use and an understanding of the process as it pertains to their clinical practice. Through answering the questions and making commitments, the EPID will prove to be much more than a simple film replacement.

## Appendix